# Editorial: Crop Breeding for Drought Resistance

**DOI:** 10.3389/fpls.2019.00314

**Published:** 2019-03-20

**Authors:** Lijun Luo, Hui Xia, Bao-Rong Lu

**Affiliations:** ^1^Shanghai Agrobiological Gene Center, Shanghai, China; ^2^Ministry of Education Key Laboratory for Biodiversity and Ecological Engineering, Fudan University, Shanghai, China

**Keywords:** drought-resistance, crop breeding, GWAS - genome-wide association study, transcriptome, phenotyping

The increased water shortage and frequent drought in agricultural ecosystems have caused tremendous problems worldwide, owning to the resulted yield losses for many crops. It is therefore essential to breed water-saving and drought-resistant crops to ensure world food security. Great progress has been made in the last decade in plant drought-resistance because of the novel findings and fast development of many new techniques and methodologies. However, accumulated knowledge about drought-resistance in crops is quite limited so far, particularly on the following questions: (1) How does drought-resistance evolve in crop during domestication; (2) How to identify drought-resistance genes and assess their potentials in breeding; (3) How to bridge the gap between theoretical research and crop breeding. To address these scientific questions, we need to establish the research topic, aiming to reveal the genetic, epigenetic, transcriptomic, and metabolomic bases of any trait associated with drought-resistance in crops, which can be applied in crop breeding.

During the last year or so, we have received more than 30 manuscripts focused on this topic and selected 13 research articles and two review articles for publication after rigorous peer review. These articles reported studies on eight crops, including rice, maize, barley, durum wheat, winter wheat, chickpea, cotton, and sweet pepper. Due to common interests in drought-resistance of different crops, any finding in a crop may provide informative references for another crop. We hope that studies on drought-resistance in different crops on this topic could benefit each other. Meanwhile, these articles apply various methodologies, including breeding and selection in the field, QTL mapping or genome wide association study (GWAS), comparative transcriptome, precise high-throughput phenotyping, and characterization of the drought-resistance genes, to study drought-resistance in different crops. To the benefit of our potential readers, we highlighted the key points of the 15 contributed articles in this research topic as follows:

Root architecture is the most promising characteristic for drought avoidance to be used in breeding. Such characteristics can greatly improve drought-resistance of crops by introducing or manipulating a single gene (e.g., *DRO1*). Lou et al. investigated transcriptomic divergences between deep-rooting and shallow-rooting rice genotypes. Based on the consistent results from a series of analyses and experimental validation, they conclude that the ATP synthesis should be a key factor to influence the root architecture. Their finding is novel and can provide valuable cues for other researchers to study genetic bases of deep-rooting in rice.

Identification of drought-resistance QTLs is essential to provide valuable targets in crop breeding. Li et al. identified four genetic regions containing SNPs significantly associated with several different traits in chickpea under drought by GWAS. This result indicated pleiotropic effects of drought-resistance associated QTLs. Gudys et al. identify 11 candidate QTLs of physiological and biochemical traits associated with drought-tolerance in Barley on a high-density function map. They further prioritize 143 candidate genes by their potential involvements in certain biological processes based on Gene Ontology annotation. Meanwhile, Cui et al. provided a new method to identify QTLs for drought-tolerance. They compared the allele frequency between drought-resistant introgression lines (resulted from strong selection in the field) and random populations and identified 13 major QTLs of drought-tolerance using the joint segregation distortion method. The most exciting result from their study is that the detected large-effect QTLs locate upstream of the genetic networks as putative regulators, which means that these QTLs could contribute significantly to drought-tolerance in breeding. In addition, they also suggest the designed QTL pyramiding strategy that is feasible for improving drought-tolerance in rice breeding.

One common difficulty for researchers to study drought-resistance is the precise evaluation of drought-resistance under field condition for a great number of genotypes. It is therefore essential to involve a high-throughput phenotyping technology in studying drought-resistance, which has drown researchers' attentions recently. Condorelli et al. described a high-throughput phenotyping platform by which the Normalized Difference Vegetation Index (NDVI) was used to precisely estimate drought-resistance traits in 248 durum wheat genotypes. Meanwhile, dozens of NDVI-based QTLs related to drought-resistance were identified by GWAS, indicating that this high-throughput phenotyping platform is valuable in the theoretical research and breeding practices.

The ABA-dependent pathway is very important for plants in responses to drought. In this volume, five articles characterized seven drought-resistance genes (*OsJAZ1, GhPLY9-11, OsDRAP1, ZmPLY8/9/12*, and *OsNAC14*) in various crops (Fu et al.; Liang et al.; He et al.; Huang et al.; Shim et al.) These genes could improve drought-resistance at seedling stage in different crops or transgenic *Arabidopsis*. However, the authors did not mention impacts of these genes on yield under drought. It is common that genes showing drought-tolerance only have minor effects in field. We consider pyramiding drought-tolerance genes, which may be a solution for the applications of these genes in production. However, such a strategy requires deep understanding in interactions among drought-resistance genes.

We appreciate the contribution from Lopes et al. who conduct large-scale field experiments to test the genotype-environment interactions in winter wheat in different experimental locations and seasons. Based on the results, they suggest that the narrow range of variation for phenology in families may facilitate the discovery and selection of new drought-resistant and -avoidant wheat lines targeting specific locations. Their proposal is valuable for the improvement of drought-resistant winter-wheat varieties.

Direct-seeding is an important method for water-saving agriculture in rice. Zhao et al. identify 13 of deep-sowing tolerance QTLs related to mesocotyl length by Non-syn GWAS (GWAS using non-synonyomous SNP). Although it is not directly related to drought-resistance, deep-sowing tolerance has a particular significance in promoting water-saving and drought-resistance rice production in China.

In one review article, Nepolean et al. reviewed the proceedings of research and breeding in maize drought-resistance, referringto precise phenotyping, genetic resources, breeding systems, drought-resistance genes, breeding informatics, etc. In the section of “MAIZE GENOMIC RESOURCES,” the authors emphasized the importance of genetic resources for drought-resistance improvement. In another review article, Ding et al. discussed the cross-talk between drought-resistance, water transport, and nitrogen uptake in higher plants. They suggest that a good management of ammonium fertilization in the field could promote water-saving agriculture and improve drought-resistance in rice. In fact, potential associations between nutrition and drought-resistance become a hot spot recently, which is also discussed in the research article by Serret et al. It requires the efforts from both biologists and agronomists to determine the associations.

From the above contributions of this research topic, some valuable cues for better understanding of drought-resistance can be found. We hope that the knowledge can facilitate further success of new studies and breeding for drought-resistance crops. For better understanding of drought-resistance in crops, combined efforts among theoretical research, variety improvement, and field management are required. Finally, we greatly appreciate the efforts of the journal editors, peer reviewers, and authors. This volume would not be available without their great contribution. We hope that our readers can identify valuable information from this volume and also find appropriate collaborators to promote their great success.

## Author Contributions

All authors listed have made a substantial, direct and intellectual contribution to the work, and approved it for publication.

### Conflict of Interest Statement

The authors declare that the research was conducted in the absence of any commercial or financial relationships that could be construed as a potential conflict of interest.

